# Medical education in the United States: do residents feel prepared?

**DOI:** 10.1007/s40037-015-0194-8

**Published:** 2015-07-17

**Authors:** Chen (Amy) Chen, Dylan Kotliar, Brian C. Drolet

**Affiliations:** 1 Harvard Medical School, 25 Shattuck St, Boston, MA 02115 USA; 2 Rhode Island Hospital, 2 Dudley St, COOP 500, Providence, RI 02903 USA

**Keywords:** Medical education, National survey, Preparation for residency

## Abstract

**Background:**

Medical schools face a growing challenge in providing a comprehensive educational experience. Students must graduate with not only the medical knowledge but also the requisite skills to care for patients and serve as physicians-in-training.

**Objective:**

To assess whether residents felt prepared by their medical school training.

**Method:**

We developed a questionnaire to assess resident attitudes towards various aspects of their medical school training and electronically distributed it among 107 United States training institutions.

**Results:**

A total of 2287 residents responded. Overall, a majority (53.8 %) agreed that ‘*medical school prepared me well to be a resident.*’ Most residents felt very well or mostly prepared in medical knowledge and clinical skills such as collecting a history (92.3 %), presenting a physical exam (86.1 %), or pathophysiology (81.6 %), but not for applied medical and psychosocial practices including end-of-life care (41.7 %), dealing with a patient death (46.3 %), and considering cost-effective care (28.7 %). Additionally, many residents reported feeling underprepared for time and fatigue management, debt, and medical-legal issues.

**Conclusions:**

Medical school graduates generally feel well prepared for residency. However, they may be less prepared to face important psychosocial, cultural and professional issues. Ultimately, a greater emphasis on skills and psychosocial experience may yield graduates who feel better prepared for today's residency challenges.

## Introduction

Resident physicians function in unique roles as both doctors and trainees. Despite having graduated from medical school, residents do not possess the experience to practice medicine independently. Yet, with more than 100,000 resident physicians in the United States (US), this group is vital in caring for patients and providing services for the functioning of much of the US health care system. Therefore, it is essential that medical school graduates are prepared for both the clinical and psychosocial aspects of the ‘residency experience’ in order to provide safe and high-quality care to patients.

Medical school serves as a critical bridge between college and graduate medical education (i.e., residency). However, medical schools face numerous challenges in providing both a comprehensive and diverse educational experience in a relatively brief time [1, 2]. While scientific subject areas have grown increasingly complex, greater educational emphasis has also been directed towards preventive medicine, end-of-life care, and other humanistic and social subjects [3, 4]. These various and growing curricular needs represent challenges for undergraduate medical educators in preparing students for the multifaceted work environment of residency and ultimately in producing competent and compassionate physicians.

Whenever possible, reform to medical school education should be empirically based. However, relatively little is known about how medical school graduates feel regarding preparation for work and life as residents. Although the United States Medical Licensing Examinations assess scientific and clinical knowledge using standardized testing, other professional and psychosocial competencies that are necessary for graduates to succeed as residents in the complex health care system are largely neglected by these exams. Similarly, the 13 core Entrustable Professional Activities put forth by the Association of American Medical Colleges as prerequisites for all new interns largely focus on clinical skills such as history-taking, physical exam performance, data interpretation, and oral presentations with little mention of psychosocial issues such as coping with patient death, fatigue mitigation strategies, or practising cost-effective care [5]. As the landscape of health care delivery shifts (e.g., increasing financial considerations, duty hour reforms, etc.), undergraduate medical education should evolve accordingly to ensure that graduates are prepared for the challenges of residency and eventually independent practice.

To provide an empirical basis for improving medical education, it is necessary to identify deficiencies in medical education. Yet, only a few small studies have attempted to assess how medical students and graduates feel regarding their preparedness for residency [6–8]. The findings of these studies were generally negative, with the majority of students reporting dissatisfaction with their undergraduate medical training. At the same time, the majority of respondents in these studies felt that they had not been taught professional skills to sufficiently prepare them for residency.

## Methods

We sought to create a descriptive evaluation of resident perceptions using a national sampling method and a self-assessment instrument that included various aspects of training such as basic sciences, clinical skills, professional and psychosocial skills, and daily work-life experience. After identification of key variables and question development, the survey was piloted with groups of residents and iteratively revised for clarity and relevance using best practices in survey design [9].

Following approval by the Institutional Review Board of Harvard Medical School, the electronic survey was sent through a request to all designated institutional officials (DIOs) at Accreditation Council for Graduate Medical Education accredited sponsoring institutions. The DIOs who agreed to participate distributed the anonymous, electronic, self-administered survey to all house officers at their respective institutions. This produced an estimated sample of 13,828 US medical graduates at 107 institutions in 36 states.

## Results

A total of 2287 US medical graduates responded to the survey (return rate 16.5 %). This sampling was generally representative of all US residents based on gender and speciality groups (Table 1). All statistically significant differences noted in the analysis remained significant (*p* < 0.05) when the tests were restricted to respondents within each speciality, gender, year in residency, and medical school setting listed in Table 1.

**Table 1:** Demographic characteristics of respondents and comparative ACGME data for all resident physicians in the United States

Characteristic	Survey Respondents Number (%)	All US Residents %^a^
**Sex**		
Male	1116 (48.8)	50.2
Female	1155 (50.5)	42.7
Not reported	16 (0.7)	7.1
**Post-graduate level of training**		
PGY-1	624 (27.3)	36.6
PGY-2	489 (21.4)	28.4
PGY-3	482 (21.1)	25.2
PGY-4	319 (13.9)	7.6
PGY-5	191 (8.4)	2.0
PGY-6	105 (4.6)	0.2
PGY-7 or greater	65 (2.8)	0.1
Not reported	12 (0.5)	–
**Type of allopathic medical school**		
Academic medical centre	2030 (88.8)	–
Community based	244 (10.7)	–
Not reported	13 (0.6)	–
**Post-graduate level of training**		
Anaesthesiology	97 (4.2)	5.6
Dermatology	44 (1.9)	1.2
Emergency medicine	198 (8.7)	4.7
Family medicine	213 (9.3)	9.0
General surgery	170 (7.4)	7.3
Internal medicine	408 (17.8)	28
Neurology	59 (2.6)	2.3
Obstetrics/Gynaecology	98 (4.3)	4.3
Pathology	58 (2.5)	2.1
Paediatrics	247 (10.8)	9.9
Psychiatry	118 (5.2)	5.3
Radiology	126 (5.5)	5.3
Surgery or surgical subspeciality	243 (10.6)	15.9
Transitional	22 (1.0)	1.1
Other	173 (7.6)	5.3
Not reported	13 (0.6)	–

*NA* not applicable, *PGY* postgraduate year.

^a^Data for US resident physicians taken from the ACGME Data Resource Book; percentages may not total % because of rounding.

Overall, about half of residents (*n* = 1230, 53.8 %) agreed with the statement *‘medical school prepared me well to be a resident.’* The large majority of residents felt very well or mostly prepared in medical knowledge and clinical skills such as collecting a history (*n* = 2111, 92.3 %), presenting a physical exam (*n* = 1969, 86.1 %), or pathophysiology (*n* = 1866, 81.6 %). In contrast, significantly fewer residents felt very well or mostly prepared for various applied clinical, professional, or psychosocial aspects of residency training (*p* < 0.001, Wilcoxon signed-rank test for all comparisons) including providing end-of-life care (*n* = 954, 41.7 %), dealing with a patient death (*n* = 1059, 46.3 %), and practising cost-effective care (*n* = 656, 28.7 %). Additionally, many residents reported feeling underprepared for debt, time and fatigue management, and medical-legal issues (Table 2). Despite these concerns, most residents reported satisfaction (i.e., mostly or very satisfied) with both career (*n* = 1848, 80.8 %) and speciality choice (*n* = 2040, 89.2 %). More than half of the residents were mostly or very satisfied with their overall quality of life (*n* = 1455, 63.6 %) and day-to-day happiness (*n* = 1537, 67.2 %), despite lower satisfaction reported with control of personal time (*n* = 924, 40.4 %) and income (*n* = 869, 38.0 %).

**Table 2:** Percentage of resident responses to each survey question

	Not at all	A little	Somewhat	Very well	Mostly
**How well did medical school prepare you for the following skills?**					
Consulting resources e.g., published articles, textbooks	1.6	6.2	23.3	32.0	36.9
Addressing medical-legal issues	8.5	26.0	34.2	8.9	22.4
Addressing social or cultural issues in providing care	1.2	6.2	21.3	35.7	35.5
Considering cost-effectiveness	13.3	26.5	31.5	9.8	18.8
Practising preventative medicine	2.1	10.4	28.0	21.4	38.1
Providing end-of-life care	7.0	19.5	31.8	15.4	26.3
**How well did medical school prepare you for the practical application of the following knowledge or skills?**					
Anatomy	1.5	10.6	22.8	27.3	37.8
Collecting a history	0.3	1.4	6.0	64.6	27.7
Laboratory diagnosis	1.2	7.5	23.2	22.6	45.4
Pathophysiology	0.3	2.9	15.2	34.4	47.2
Pharmacology	2.4	13.1	28.6	17.3	38.6
Physical diagnosis	0.7	4.7	19.3	29.8	45.4
Presenting a history and physical	0.6	3.9	9.5	53.6	32.5
**How well did medical school prepare you for:**					
Dealing with an angry patient	7.3	21.1	30.4	13.4	27.8
Dealing with patient death	6.6	15.1	32.0	18.5	27.9
Debt and financial management	16.3	25.8	30.2	8.2	19.4
Developing a rapport with patients	1.1	3.6	15.9	43.8	35.6
Fatigue management	12.5	21.4	32.0	10.7	23.4
Time management	4.4	11.7	27.7	21.5	34.6
**On most days, how satisfied are you with:**					
Your choice of speciality	1.1	2.5	7.2	32.2	57.0
Your choice to become a physician	1.7	5.1	12.5	37.6	43.2
Your lifestyle outside of work	5.8	13.2	27.1	17.7	36.2
Control of your personal time	11.0	19.5	29.1	11.8	28.6
Your income	13.0	19.5	29.6	9.5	28.5
Your overall quality of life	3.6	9.0	23.8	17.3	46.3
Your day-to-day happiness	3.5	8.0	21.3	20.2	47.0
	**Disagree**		**Neutral**		**Agree**
**Please indicate your level of agreement:**					
Medical school prepared me to be an excellent resident	9.8		36.4		53.8

## Discussion

In this large study of US medical graduates, we found that more than half of the respondents (53.8 %) felt that they were ‘well prepared’ by medical school to be a resident. For those with a more negative overall perception, the sentiment may be due to feeling underprepared specifically for psychosocial and professional aspects of residency, especially since most residents reported feeling well prepared for the basic science and clinical proficiencies assessed by the survey. More specifically, the data demonstrates that many US medical graduates do not feel sufficiently prepared to deal with some of the challenges of residency and modern medical practice including fatigue, debt management, end-of-life care, dealing with angry patients, providing cost-effective care, and confronting medical-legal issues.

As with all aspects of training, some education in these areas should occur in medical school while other training will occur on the job during residency. However, the time constraints of residency are equally or perhaps more challenging than those of medical school; therefore, it is worth considering whether some attention should be focused on these weaknesses during undergraduate medical education. Of the ‘weak areas’ identified in the survey, coping with patient suffering and death, providing quality end-of-life care, and practising cost-effective care are good examples of subjects that might benefit from more focused and longitudinal exposure during medical school.

The ability to cope with patient suffering and death is critical to maintaining healthy and compassionate physicians while preventing burnout [10]. Burnout is a well-recognized problem in the medical profession and is encountered at much higher rates than in the general population, along with other psychiatric illnesses including substance abuse and depression [11–14]. Beyond the obvious and important implications of psychiatric illness in medical professionals, studies have found that physician burnout is associated with decreased patient satisfaction and increased likelihood of reported medical errors [13–15]. Providing medical students with tools to better prepare them for the challenges of residency may help to address some of these critical issues [16].

We also found that fewer than half of all residents surveyed felt prepared to provide end-of-life care (*n* = 954, 41.7 %). Yet, proficiency in addressing end-of-life needs along with providing basic palliative care is essential to quality health care delivery [17]. While these skills can be honed through on-the-job experiences, in-depth background and longitudinal exposure during medical school could substantially improve this necessary skill. This type of training could be implemented through an integrated end-of-life and palliative care education over four years. As an example, in year 1 of Yale School of Medicine’s new curriculum, students participate in an ‘Introduction to end-of-life care workshop’ that includes recurrent interviews of a patient with a terminal illness. In years 2 and 3, they visit inpatient or home hospice centres to observe patient care, conduct guided interviews, and write self-reflections and case write-ups. Finally, during year 4, they engage in a terminal illness workshop and a class on resuscitation orders, death pronouncement, and notification. Such a longitudinal experience is a model of how medical schools could better prepare their trainees to cope with patient suffering and death while providing end-of-life care before residency when the practical and acute application of these skills is necessary. Although the efficacy of this model could not be tested by subset analysis of this anonymized survey, graduates with this background could make an interesting intervention group for future studies.

Finally, less than one-third of residents (*n* = 636, 27.8 %) felt prepared to practise cost-effective medicine. With health care spending projected to nearly double by 2022 and an estimated depletion of Medicare funds by 2029, the ability to provide cost-effective care is well recognized as an essential aspect of health care delivery [18–20]. Awareness of efficiency and providing cost-effective care are learned skills that require early training and new mindsets that emphasize providing the best possible care to the individual patient while maintaining a global perspective on community health through optimal resource utilization [21]. Such thinking could be cultivated in medical students by including aspects of cost-effectiveness among the discussions of diagnostic and treatment strategies already covered in much of case-based learning and clinical clerkships. Additionally, higher-level discussions about topics such as ethical considerations, preventative care, and insurance systems could be included in short health care policy seminars already occurring in some medical schools.

## Limitations

The major limitation of this survey is the relatively low response rate, and therefore the potential for confounding selection bias. However, the demographics of our survey respondents generally match those of all US residents and conclusions were consistent across demographic categories, which diminishes our concern for selection bias. A second major limitation is the uncertainty in hypothesis testing. Although statistically significant differences were noted among the responses to most variables, the magnitude of the difference between positive and negative responses was sometimes small. As such, the results should be interpreted from a descriptive standpoint, e.g. representing a large (plurality rather than majority) group of residents reporting lack of preparedness in such areas as described. Finally, this survey research is unable to assess the reasons for residents’ reported perceptions, and a worthwhile future direction could include focus groups and interviews of residents regarding their medical school education. In particular, educators could benefit from more thorough analyses of how and why residents feel more or less prepared for each of the various aspects of the residency experience evaluated in this survey.

## Conclusion

Based on this descriptive study from a national sample of US residents, about half of medical school graduates feel well prepared to enter the work force as physician-trainees. The data suggest that residents may feel less prepared to face important psychosocial, personal and professional issues such as end-of-life care, dealing with angry patients, cost-effective decision-making, and debt management. To this end, undergraduate medical educators may consider refocusing some curricular efforts on highlighting these topics to cultivate prerequisite knowledge and provide a greater diversity of experiences before the beginning of residency. Ultimately, improvements in these areas may yield graduates who feel better prepared for the challenges of today’s residency training environment.

### Essentials

- Medical school serves as a critical bridge between college and residency, preparing residents to function in their unique roles as both doctors and trainees.
- Only a few small studies have attempted to assess how prepared medical students and graduates feel for their experience as residents, and the findings of these studies were generally negative.
- Through a national survey of US residents, we found that medical school graduates generally feel well prepared for residency, but feel less prepared in certain important psychosocial, cultural and professional aspects.
- A greater emphasis on skills and psychosocial experience in medical school may yield graduates who feel better prepared for today’s residency challenges.
